# Rapid and dose-dependent increase of 25(OH)D levels after calcifediol supplementation in a woman with obesity, chronic liver disease, and osteoporosis

**DOI:** 10.20945/2359-4292-2024-0428

**Published:** 2025-10-08

**Authors:** Gustavo Kendy Camargo Koga, Sergio Setsuo Maeda, Marise Lazaretti-Castro

**Affiliations:** 1 Departamento de Medicina, Escola Paulista de Medicina, Universidade Federal de São Paulo, São Paulo, SP, Brasil

**Keywords:** Calcifediol, cholecalciferol, vitamin D deficiency, dosage

## Abstract

VitaminD deficiency is a global concern, and calcifediol serves as an alternative to cholecalciferol for achieving and maintaining optimal vitamin D levels, despite the lack of international guidelines for calcifediol supplementation regimens. We present a case involving a 58-year-old patient with osteoporosis and a medical history of type 2 diabetes, obesity, and cirrhosis. Standard treatment with calcium, cholecalciferol, and bisphosphonate was initiated; however, supplementation failed to achieve the target vitamin D levels during follow-up. Subsequently, calcifediol was introduced at a dose of 10 mcg daily, which was increased to 20 mcg daily after one month. Nonetheless, the vitamin D serum concentration rose to 80 ng/mL by the third month, prompting discontinuation of the drug and levels gradually decreased to 28 ng/mL over 2.5 months. Upon the administration of calcifediol at 10 mcg three times a week, serum levels stabilized at 35 ng/mL. Calcifediol offers several advantages over cholecalciferol, including better intestinal absorption, bypassing the need for hepatic hydroxylation, and a more rapid increase in 25-hydroxyvitamin D (25[OH]D) levels. Current guidelines recommend considering calcifediol in cases of obesity, malabsorption syndromes, and chronic hepatic diseases, although optimal dosages remain uncertain. Based on the commercially available tablet in Brazil, we suggest initiating calcifediol at 10 mcg per day and adjusting the dose according to 25(OH)D levels.

## INTRODUCTION

Vitamin D deficiency is a global health concern, and serum 25-hydroxyvitamin D (25[OH]D) concen-trations are utilized to assess vitamin D status (^1^). It is estimated that 37.3% of the global population exhibits vitamin D deficiency (25[OH]D < 20 ng/mL) (^2^), and a meta-analysis of 340,476 Brazilians revealed a prevalence of 28.2% (^3^).

Maintaining optimal vitamin D levels is crucial for bone metabolism, musculoskeletal health, and poten-tially other extra-skeletal functions. Its deficiency can lead to rickets, osteomalacia, and secondary hyperparathyroidism, while also increasing the risk of falls and fractures (^4^). Consequently, achieving vitamin D levels above 30 ng/mL is a standard in osteoporosis treatment (^5-7^).

The most common approach to adjusting 25(OH)D concentrations involves supplementation with vitamin D itself. In Brazil, this is available as cholecalciferol (vitamin D_3_), while in countries such as the USA and India, ergocalciferol (vitamin D_2_) is also used (^8^).

Recently, calcifediol (25[OH]D) has been proposed as an alternative to cholecalciferol. Although it has been commercially available in Spain for over 40 years (^9^), few studies have explored the pharmacological characteristics of this drug, which has recently gained relevance following positive outcomes during the COVID-19 pandemic (^10^).

Calcifediol has been approved by the Brazilian Health Regulatory Agency for use as a supplement since 2022. Nonetheless, there remains a lack of Brazilian and international guidelines recommending the most appropriate supplementation schemes (^9,11,12^).

Considering the advantages of calcifediol in specific situations, we present our initial experience with calcifediol in a woman with osteoporosis, chronic liver disease, obesity, and hypovitaminosis D, who failed to achieve 25(OH)D concentrations of 30-60 ng/mL despite cholecalciferol administration. The objective is to advocate calcifediol as an alternative for patients with certain comorbidities, highlight the rapid increase in 25(OH)D levels, and alert the medical community about the risks of supraphysiological levels with higher doses and the need for individual adjustments, framing this case as exploratory.

## CASE REPORT

A 58-year-old woman was evaluated for subclinical hypothyroidism. Her medical history revealed type 2 diabetes, overweight defined by a body mass index of 29.1 kg/m^2^, and child A cirrhosis due to non-alcoholic fatty liver disease. Routine tests are presented in **Table 1** and revealed thrombocytopenia, slight alterations in liver function, and a 25(OH)D level of 21.4 ng/mL. Dual-energy X-ray absorptiometry demonstrated osteoporosis, indicated by a T-score of the lumbar spine of -2.8 standard deviations.

**Table 1:** Patient biochemical measurements over time

	First evaluation	Before calcifediol	Months after calcifediol	Reference values
Serum calcium (mg/dL)	10.1	9.3	9.8	8.6-10.2
Albumin (g/dL)	4.8	4.6	4.4	3.5-5.2
Ionized calcium (nmol/L)	1.33	-	-	1.24-1.41
Phosphorus (mg/dL)	-	2.9	2.9	2.5-4.5
PTH (pg/mL)	45.8	31.6	53.0	15-65
24-h UCa (mg/24 h)	-	-	166.3	100-321
Serum creatinine (mg/dL)	0.72	0.73	0.75	0.5-0.9
Hemoglobin (g/dL)	14.4	13.7	14.2	12-15.5
Platelets (x10^3^/µL)	140	97	90	150 -450
AST (U/L)	54	32	42	<32
ALT (U/L)	59	25	33	<33
GGT (U/L)	121	47	-	<40
Alkaline phosphatase (U/L)	97	99	95	35-105
INR	1.01	0.93	-	(1-1.2)
Total bilirubin (mg/dL)	0.86	0.44	1	<1

ALT, alanine aminotransferase; AST, aspartate aminotransferase; GGT, gamma-glutamyl transferase; INR, international normalized ratio; PTH, parathyroid hormone; 24-h Uca, 24-hour urinary calcium.

She had no history of fragility fractures or glucocorticoid use and became postmenopausal at age 51. Alendronate was prescribed, and cholecalciferol was optimized to 350 mcg (14,000 IU) once a week, equivalent to 50 mcg/day. Her dietary calcium intake was adequate (1,200 mg/day).

During follow-up, her weight increased (body mass index of 30.9 kg/m^2^), and she demonstrated poor medication adherence. Alendronate was replaced with zoledronate following an episode of upper gastrointestinal bleeding due to esophageal varices.

**Figure 1:**
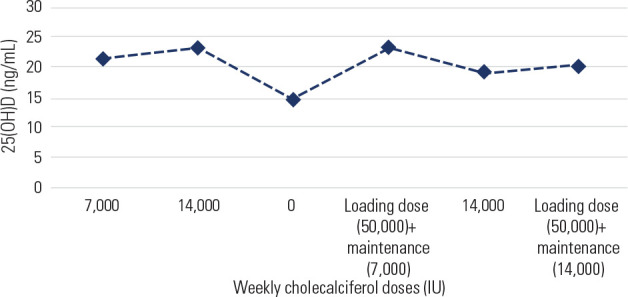
25-hydroxyvitamin D [25(OH)D] concentrations over time with concomitant cholecalciferol doses.

Throughout the COVID-19 pandemic, she lost follow-up and experienced a tibial fragility fracture. Upon return, calcium carbonate was initiated, and a second dose of zoledronate was administered. One year later, poor adherence was noted, as she had discontinued cholecalciferol use, which could explain her deficiency at the onset (14.6 ng/mL). However, adherence information in the latest data is unavailable, and she maintained suboptimal 25(OH)D levels despite prescribed loading doses (cholecalciferol 1,250 mcg [50,000 IU] weekly for eight weeks) and maintenance of 350 mcg (14,000 IU) weekly (**Figure 1**).

25(OH)D: 25-hydroxyvitamin D

In 2023, her 25(OH)D levels remained below 30 ng/mL. At this point, calcifediol became available in Brazil. Given her difficulty achieving the target 25(OH)D level of 30-60 ng/mL (**Figure 1**) and her history of chronic liver disease and obesity, calcifediol was initiated at 10 mcg per day for the first month and increased to 20 mcg per day thereafter, to evaluate vitamin D behavior at different doses of calcifediol, as recommended by some guidelines (^13,14^). Consequently, blood samples for 25(OH)D levels were collected monthly.

**Figure 2:**
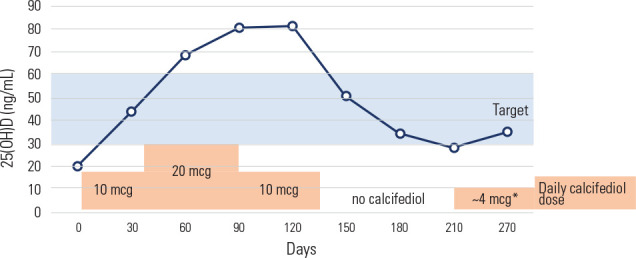
Evolution of 25-hydroxyvitamin D [25(OH)D] levels during follow-up with different calcifediol doses.

After one month of calcifediol at 10 mcg/day, her 25(OH)D level was on target; however, at 20 mcg/day, the 25(OH)D concentrations exceeded 60 ng/mL (**Figure 2**). Calcifediol was subsequently reduced to 10 mcg/day, but after one month, the 25(OH)D level remained at 81.1 ng/mL, prompting the discontinuation of calcifediol. No hypercalcemia or acute renal injury was observed (**Table 1**). Subsequently, serum 25(OH)D le-vels progressively decreased to 28 ng/mL, as shown in **Figure 2** (approximately 2.5 months after discontinuation), at which point calcifediol was restarted.

* Calcifediol 10 mcg administered 3 times per week (approximately equivalent to a daily dose of 4 mcg)

25(OH)D: 25-hydroxyvitamin D

## DISCUSSION

Vitamin D deficiency is linked to impaired bone health and is commonly observed in conditions such as obesity and chronic liver disease. In patients with osteoporosis, the goal is to maintain 25(OH)D levels of 30-60 ng/mL (^5^). However, cholecalciferol was ineffective in achieving adequate vitamin D levels in this patient, despite an initial loading dose of 50,000 IU weekly for 8 weeks, followed by a maintenance dose of 14,000 IU per week.

Thus, calcifediol may be an alternative to optimize 25(OH)D status and should be considered in specific situations, including obesity, malabsorption syndromes (*e.g.*, Crohn’s disease, celiac disease, and cystic fibrosis), bariatric surgery, chronic liver diseases, and the use of drugs that interfere with the hepatic cytochrome P-450 enzyme system, such as corticosteroids, anticonvulsants, and antiretrovirals (^8,11,15^).

Calcifediol has a higher rate of intestinal absorption in healthy individuals compared to cholecalciferol (93% vs. 79%) (^16^). As a lipophilic steroid, cholecalciferol’s absorption depends on the presence of bile acids and micellar formation (^8,16^). Inside the intestinal cells, vitamin D binds to chylomicrons and is transported through the lymphatic system into the bloodstream (^16,17^).

In contrast, calcifediol is readily absorbed by enterocytes and transported via the portal vein into the general circulation (^16,17^), resulting in a higher plasma peak after oral ingestion and greater overall bioavailability (^16^). Additionally, calcifediol may be beneficial in situations of intestinal malabsorption, as its absorption is nearly unchanged or only slightly reduced, whereas cholecalciferol’s absorption is significantly compromised (^8,16,17^).

The structure of calcifediol includes an additional hydroxy (OH) group, which makes it more polar and hydrophilic (^8,17^). This reduces its tendency to accumulate in adipose tissue (^8,15^) and increases its affinity for the vitamin D binding protein (^18^). Furthermore, calcifediol does not require hepatic hydroxylation by the enzyme 25-hydroxylase (*CYP2R1*), which may have reduced activity in liver diseases and obesity (^8,16^), as seen in this patient.

Considering its superior absorption efficacy and the lack of a need for hepatic conversion, calcifediol has a more predictable linear dose-response curve, independent of baseline 25(OH)D levels (^8,16,19^). Long-term treatment with calcifediol results in stable and sustained 25(OH)D concentrations (^12^).

Therefore, these two molecules exhibit different pharmacokinetics (^20^), and their doses are not equivalent (^21^). Calcifediol is more potent than vitamin D_3_, increasing 25(OH)D levels 3 to 6 times more efficiently, which leads to a quicker and greater increase in 25(OH)D concentration (^8^). A study involving twenty healthy postmenopausal women found that those supplemented with 20 mcg (800 IU) of cholecalciferol achieved a maximum mean 25(OH)D concentration of 31 ng/mL after 3-4 months. Conversely, the group receiving 20 mcg of calcifediol reached 69.5 ng/mL within the same period, with all participants attaining levels above 30 ng/mL after 35 days (^22^).

In another study involving forty postmenopausal osteopenic women, the group receiving 20 mcg of cholecalciferol (800 IU) increased their 25(OH)D levels from 16.2 to 32 and 34.5 ng/mL after 6 and 12 months, respectively. The group receiving 20 mcg of calcifediol daily raised their levels from 14.9 to 64.4 and 75.2 ng/mL in the same time frames (^21^). Calcifediol has a faster elimination rate than cholecalciferol (^8,23^) and a shorter half-life, with 10-15 days compared to 2 months, respectively (^8,20^). Upon discontinuation, 25(OH)D levels quickly return to baseline (^12^). In our single-case study, 25(OH)D concentrations decreased from 81.1 to 50.5 ng/mL after 13 days of suspension.

Calcifediol appears to be safe, with a low risk of toxicity (^9,11,24^), although safety data are limited, and most studies involve small sample sizes (^11^). Despite being rare (^8^), there is a risk of toxicity when overdoses are used over extended periods, particularly due to prescription errors or patient self-administration (^20^). The risk of intoxication is considered when 25(OH)D levels exceed 100 ng/mL (^5^), which can lead to hypercalcemia, hypercalciuria, and nephrocalcinosis (^8^).

In Spain, there have been few reports of intoxication in over 40 years of calcifediol use (^8^). In 2019, the Spanish Agency for Medicines and Health Products issued a warning about serious cases of hypercalcemia in adults related to calcifediol, which were associated with higher dosage frequencies than recommended (^25^).

Despite observing supraphysiological 25(OH)D concentrations, the levels remained below 100 ng/mL, and calcium, urinary calcium, and creatinine levels persisted within reference values. No evidence supports levels above 60 ng/mL (^5^), which are associated with an increased risk of falls in postmenopausal women (^26^).

The prescribed dosage, frequency, and duration depend on baseline 25(OH)D levels, patient characteristics, the condition being treated, and comorbidities (^8^). Nonetheless, few international guidelines recommend specific calcifediol regimens. Some recommendations for treating vitamin D deficiency do not mention the use of calcifediol (^7,27,28^). However, the Central and Eastern European Expert Consensus Statement suggests that calcifediol may be considered for patients with obesity, malabsorption syndromes, and chronic hepatic or renal diseases, though it does not provide specific regimens (^29^).

Calcifediol is available in several European countries, with Spain and Italy being notable for its extensive use (^18^). The Spanish Society for Bone Research and Mineral Metabolism recommends 8-12 mcg/day or 266 mcg every 3-4 weeks for patients with osteoporosis or those at risk of vitamin D deficiency, with higher doses necessary for severe deficiency (below 10 ng/mL). They advise retesting serum concentrations every 12-16 weeks until target levels are reached (^13^).

The Italian guidelines suggest calcifediol at doses of 15-20 mcg/day or 100-150 mcg/week (^14^). In contrast, the Italian Medicine Agency recommends specific dosages based on baseline 25(OH)D concentrations: 266 mcg twice per month for levels below 12 and 266 mcg per month for levels between 13-20 ng/mL, with serum levels to be reevaluated in about 12 weeks (^30^).

Daily, weekly, and monthly administration of calcifediol appears effective (^9,11,24^), although there is no consensus on the optimal dosages. Few studies have evaluated daily use, with tested doses varying, most commonly 10 or 20 mcg; higher dosages (*e.g.*, 40 mcg/day) should be avoided (^24^).

In our single-case experience, a 10 mcg daily dose of calcifediol was initiated following the Spanish guidelines (^13^). This dose achieved optimal 25(OH)D levels, but when increased to 20 mcg, a dose recommended by the Italian guidelines (^14^), concentrations exceeded 60 ng/mL (**Figure 2**). This observation aligns with findings from other studies in the literature (^21,22^). After discontinuing calcifediol, an empirical dose of 10 mcg three times a week maintained concentrations within the target range. The testing period differed from that supported by guidelines for scientific and study purposes.

This case report has limitations. There is no record of adherence to cholecalciferol throughout the follow-up, except in the early years. Poor adherence could be a reason for not achieving therapeutic goals and should always be considered. Additionally, cholecalciferol doses higher than 14,000 IU/week were not prescribed. As an observational single-patient evaluation, these findings cannot be generalized.

Despite these limitations, this report contributes to a better understanding of calcifediol as an alternative strategy for vitamin D replacement and could guide physicians in prescribing it.

In conclusion, this case report highlights a real-world scenario where calcifediol was used in a patient with osteoporosis, liver impairment, and obesity. It can be a safe alternative to cholecalciferol, offering advantages such as a more rapid increase in 25(OH)D levels. More research is necessary to establish safe and ideal daily dosages. We suggest starting calcifediol at 10 mcg per day, as this is the available formulation in our country, with retesting after 12 weeks, according to Italian and Spanish guidelines. Individual doses should be titrated based on 25(OH)D levels to prevent excess.

## Data Availability

datasets related to this article will be available upon request to the corresponding author.
